# Elevation of SIPL1 (SHARPIN) Increases Breast Cancer Risk

**DOI:** 10.1371/journal.pone.0127546

**Published:** 2015-05-19

**Authors:** Jason De Melo, Damu Tang

**Affiliations:** 1 Division of Nephrology, Department of Medicine, McMaster University, Ontario, Canada; 2 Father Sean O’Sullivan Research Institute, Ontario, Canada; 3 The Hamilton Center for Kidney Research, St. Joseph’s Hospital, Hamilton, Ontario, Canada; INRS, CANADA

## Abstract

SIPL1 (Sharpin) or Sharpin plays a role in tumorigenesis. However, its involvement in breast cancer tumorigenesis remains largely unknown. To investigate this issue, we have systemically analyzed *SIPL1* gene amplification and expression data available from Oncomine datasets, which were derived from 17 studies and contained approximately 20,000 genes, 3438 breast cancer cases, and 228 normal individuals. We found a *SIPL1* gene amplification in invasive ductal breast cancers compared to normal breast tissues and a significant elevation of SIPL1 mRNA in breast cancers in comparison to non-tumor breast tissues. These results collectively reveal that increases in SIPL1 expression occur during breast cancer tumorigenesis. To further investigate this association, we observed increases in the *SIPL1* gene and mRNA in the breast cancer subtypes of estrogen receptor (ER)+, progesterone receptor (PR)+, HER2+, or triple negative. Additionally, a gain of the *SIPL1* gene correlated with breast cancer grade and the levels of SIPL1 mRNA associated with both breast cancer stages and grades. Elevation of *SIPL1* gene copy and mRNA is linked to a decrease in patient survival, especially for those with PR+, ER+, or HER2- breast cancers. These results are supported by our analysis of SIPL1 protein expression using a tissue microarray containing 224 breast cancer cases, in which higher levels of SIPL1 relate to ER+ and PR+ tumors and AKT activation. Furthermore, we were able to show that progesterone significantly reduced SIPL1 mRNA and protein expression in MCF7 cells. As progesterone enhances breast cancer tumorigenesis in a context dependent manner, inhibition of SIPL1 expression may contribute to progesterone's non-tumorigenic function which might be countered by SIPL1 upregulation. Taken together, we demonstrate a positive correlation of SIPL1 with BC tumorigenesis.

## Introduction

SIPL1 (Shank-Interacting Protein-Like 1), also known as Sharpin (Shank-associated RH domain interacting protein), was identified in 2001 as a Shank-binding protein in the postsynaptic density and later in 2003 was reported shown to be expressed in the gastric fundus [1,2]. SIPL1/Sharpin is a major component of an E3 ubiquitin-protein ligase complex, the linear ubiquitin chain assembly complex (LUBAC); the complex consists of HOIL-1, HOIP, and SIPL1/Sharpin, and adds a linear polyubiquitin chain to protein substrates [3–7]. The most thoroughly investigated function of SIPL1/Sharpin is the modification of NEMO, an adaptor protein facilitating NF-κB activation, via linear polyubiquitination, resulting in NF-κB activation [8]. In accordance with the essential roles of NF-κB signalling in the immune system, loss of SIPL1/Sharpin compromises a variety of immunoreactions [9–11], and causes chronic proliferative dermatitis in mice, which is largely attributable to abnormalities in the inflammatory response [3,5,7,12].

The essential contribution of SIPL1 to the activation of NF-κB support the possibility that SIPL1 promotes tumorigenesis, as NF-κB signalling possesses well-demonstrated tumorigenic properties [13]. This prospect is further supported by SIPL1/Sharpin-mediated suppression of apoptosis in keratinocytes and hepatocytes [14,15], and repression of cisplatin, a widely-used drug in cancer therapy, induced apoptosis [16]. Additionally, SIPL1 promotes the migration of CHO cells *in vitro* and lymphocytes in vivo, and enhances the lung metastasis of osteosarcoma *in vivo* (in immunocompromised mice) [10,17,18]. Upregulation of SIPL1 was observed in ovarian cancer, renal cell carcinoma, and cervical cancer [17,19,20]. Furthermore, SIPL1 was reported to inhibit PTEN via a physical interaction [20]. Collectively, evidence demonstrates a role of SIPL1 in promoting tumorigenesis.

Whether SIPL1 plays a role in breast cancer (BC) tumorigenesis remains unknown. BC is the most common malignancy diagnosed and the second leading cause of cancer-related deaths in women [21]. BC is a highly heterogeneous group of diseases, which can express ER (ER+), PR (PR+), HER2+, or none of them (ER-, PR-, and HER2-/triple negative) [22]. The HER2+ and triple negative (TN) BCs have poor outcomes [21,23] and comprise 20–25% and 10–25% of the reported cases, respectively [24–27].

To study a possible association of SIPL1 and BC tumorigenesis, we have taken advantage of the rich resources of cancer genome data and gene expression profiles deposited in the Oncomine database, and thoroughly analyzed the association of SIPL1 gene amplification and expression during BC tumorigenesis. This analysis together with our studies of the SIPL1 protein in primary BCs reveals a positive correlation of SIPL1 with BC tumorigenesis.

## Materials and Methods

### Tissue microarray immunohistochemistry

A breast cancer tissue microarray (TMA) was obtained from the Cancer Diagnosis Program (formerly the Cooperative Breast Cancer Tissue Resource; CBCTR) which is funded by the National Cancer Institute [28]. The TMA was organized to examine markers associated with BC progression, and contained 239 breast carcinomas, including 80 cases each for node negative and positive tumor tissues and 79 cases of distant metastatic BC cancers. There was no follow-up information available for these patients.

The slides were first deparaffinized and rehydrated using successive washes in Xylene and EtOH baths. The slides were then heat treated in a sodium citrate buffer (10 mM) for 20 minutes in a food steamer. The slides were blocked using a buffer containing goat serum, and incubated with anti-SIPL1 antibody (1:100) [20] or anti-AKT pS473 (1:100, Rockland Immunochemicals Inc., Gilbertsville, PA) overnight at 4°C. Biotinylated goat anti-rabbit IgG was then incubated with the slides for one-hour followed by an incubation with Avidin-Biotin Complex (ABC) for one-hour (Vector Laboratories, Burlington, ON). Chromogen detection was carried out using diaminobenzidine (DAB; Vector Laboratories, Burlington, ON) and counterstained with Haematoxylin (Sigma Aldrich, Oakville, ON). The staining was repeated in duplicate.

The slides were scanned at the Advanced Optical Microscopy Facility (AOMF) at the University of Toronto using a ScanScope. All slide images were analyzed using ImageScope software (Leica Microsystems Inc., Concord, ON). All cores were visually examined. Damaged cores were excluded, leaving 206 total cancer samples in the analysis. The intensity values obtained from the Imagescope software after analyses were converted to an HScore using the formula [HScore = (% Positive) x (intensity) + 1] [20]. The HScore was normalized using a background subtraction and averaged between the two replicates. The TMA contained MCF10A cells. Based on our western blot analysis of SIPL1 and pAKT expression in various breast cancer cells lines (data not shown), MCF10A cells were found to express low levels of both events. With this understanding, we determined the respective average HScore for SIPL1 and pAKT in the MCF10A cores of our TMA. These scores were subsequently used as a respective threshold to define the status (positive versus negative) of SIPL1 and pAKT in TMA tissues. All tissue cores classified as positive and negative were confirmed by visual inspection.

### Cell culture

MCF7 cells were obtained from American Type Culture Collection (ATCC; Manassas, VA), and cultured in DMEM media supplemented with 10% Foetal Bovine Serum (FBS; Sigma Aldrich; Oakville, ON) and 1% Penicillin-Streptomycin (Life Technologies; Burlington, ON). Prior to hormone treatment the cells were washed with PBS three times and grown in phenol-red free DMEM (GE Healthcare; Logan, Utah) supplemented with 5% dextran-charcoal treated FBS (Life Technologies; Burlington, ON); and 1% Penicillin-Streptomycin for 72 hours. The cells were treated with 10 nM 17β-estradiol (E2; Sigma Aldrich; Oakville, ON), a combination of 10 nM E2 and 10 nM progesterone (P4; Sigma Aldrich; Oakville, ON) or a vehicle control (EtOH) for 24 hours.

### Real-time PCR analysis

After treatment with the respective hormones, the cells were lysed and total RNA was collected using TRIzol (Life Technologies, Burlington, ON) following the manufacturers protocol. Reverse transcription and qRT-PCR was carried out as previously described [29]. Briefly, 2 μg of RNA was converted to cDNA, followed by qRT-PCR, where 1 μL of cDNA was used in each reaction. Real time PCR primers used for actin (Forward: 5’- ACC GAG CGC GGC TAC AG -3’; Reverse: 5’- CTT AAT GTC ACG CAC GAT TTC C -3’), SIPL1 (Forward: 5’- GCT ATT GCA GGT GGA GAC GA -3’; Reverse: 5’- GCC TCC TGA AGC TGA ACA CT -3’), BCL2 (Forward: 5’- GGT GGG GTC ATG TGT GTG G -3’; Reverse: 5’- CGG TTC AGG TAC TCA GTC ATC C -3’) and MYC (Forward: 5’- GGC TCC TGG CAA AAG GTC A -3’; Reverse: 5’- CTG CGT AGT TGT GCT GAT GT -3’).

### Western blot analysis

Western blot analysis was carried out using our established protocol [29]. Briefly, 50 μg of protein lysate was separated on SDS-PAGE gels and transferred onto Amersham Hybond ECL nitrocellulose membranes (Amersham, Baie d’Urfe, QC). Blots were blocked with 5% skim milk and incubated at 4°C overnight with either an anti-SIPL1 [20] or anti-GAPDH (1:5000, Cell Signalling, Danvers, MA). The blots were then incubated with the corresponding HRP-conjugated secondary antibodies for one hour at room temperature. Signals were detected using an ECL Western Blotting Kit (Amersham, Baie d’Urfe, QC). Protein bands were quantified using ImageJ software (National Institutes of Health).

### Oncomine

Oncomine (Compendia Bioscience, Ann Arbor, MI; www.onocomine.org) is an online database consisting of previously published and publicly available microarray data. Using the search term “SIPL1” and isolating for datasets representing Ductal Breast Carcinoma and Invasive Ductal Breast Carcinoma, we identified 21 datasets which contained DNA or RNA expression data. Four datasets did not contain information relevant to this study and were thus excluded. The detailed dataset information was exported and analyzed in terms of clinical-pathological information and SIPL1 expression. The follow-up period for these patients was up to 25 years with an average of 8–10 years.

Seven datasets contained normal breast tissue controls and these were used to compare the expression of SIPL1 in normal and cancer tissues. In order to score cancers as SIPL1 positive (high expression) or SIPL1 negative (low expression), the Log2 Median-Centered ratio, as reported in the Oncomine database, for all the normal samples in a particular dataset were averaged. RNA expression values above and below this average were considered SIPL1 positive and negative, respectively. Likewise, for the DNA copy number data, the Log2 copy number units reported in Oncomine were converted to a copy number value using the formula [2 x 2^(Log2 Copy Number Value)^]. Values above 2 were considered to be SIPL1 positive (SIPL1 amplified) and those below were considered to be SIPL1 negative (no SIPL1 amplification).

### Statistical analysis

All statistical analysis was carried out using GraphPad Prism 5 software. A *p*<0.05 was considered statistically significant.

## Results

### Amplification of the *SIPL1* gene in breast cancer

**Fig 1:**
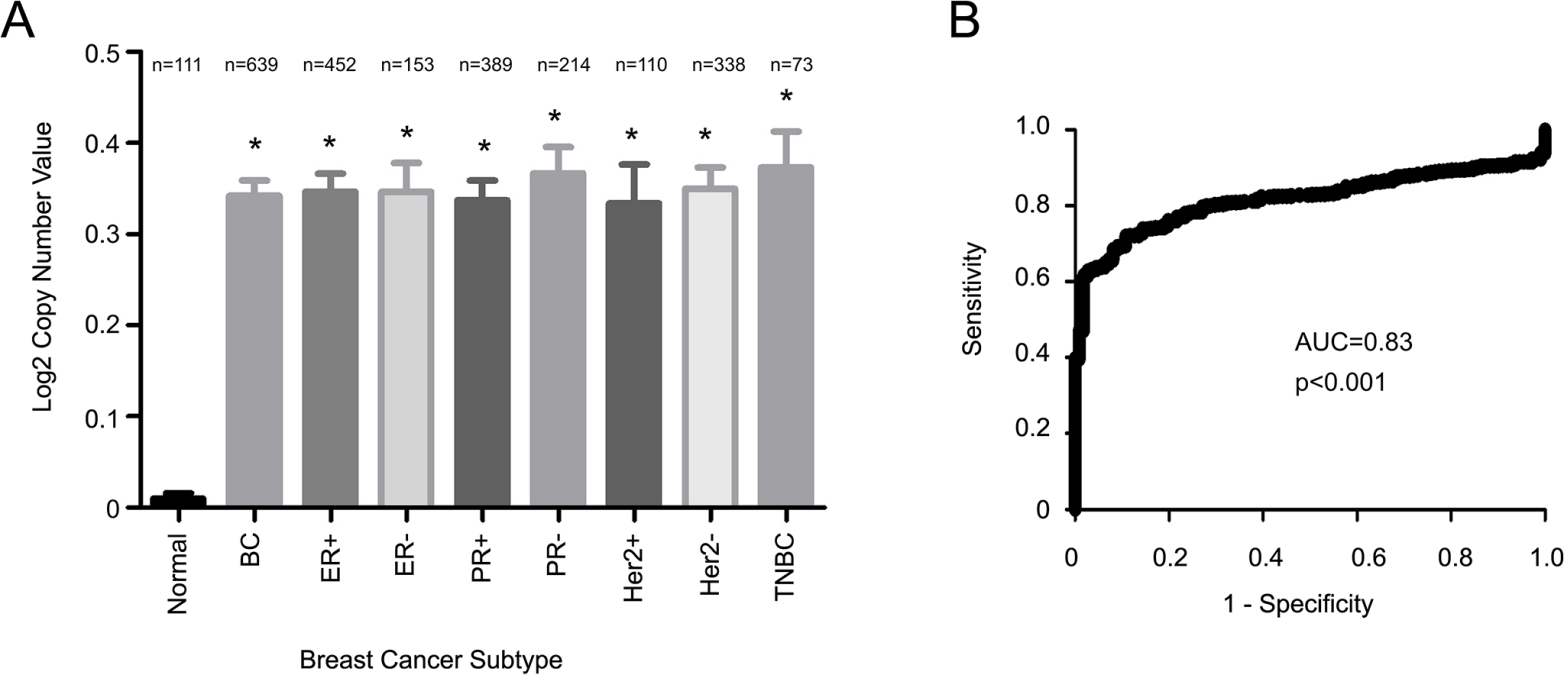
Amplification of SIPL1 in breast cancer. Data from the TCGA 2 dataset was extracted from Oncomine (Compendia Bioscience, Ann Arbor, MI) and analyzed with respect to SIPL1 gene copy number variation (GCN) in cancer vs. normal tissues. Statistical analysis was conducted using an unpaired, two-tailed, welch-corrected t-test. Asterisks indicate p<0.0001 in comparison the normal breast tissues. A Log2 copy number unit of 0 equates to a gene copy number of 2 (**A**). (**B**) A receiver-operating characteristic (ROC) curve of normal versus primary breast cancer was calculated based the data extracted from the TCGA dataset. AUC: area under the curve.

The SIPL1 gene is located at 8q24.3, a region that is gained (or amplified) in 40% of breast cancers [30–35], indicating a possible gain of the *SIPL1* gene during the course of the disease. The recent characterization of many cancer genomes has accumulated a rich source of data regarding aneuploidy, copy number variations, and somatic mutations. This information has been deposited into the Oncomine database. In taking advantage of the characterized genome for 639 breast cancers and 111 normal controls, we observed increases of the *SIPL1* gene copy number in breast cancer in comparison to normal breast tissues, and this gain was detected in all subtypes of breast carcinomas, including those of ER+, ER-, PR+, PR-, HER2+, and triple negative (Fig 1A). Additionally, receiver-operating characteristic (ROC) analysis shows that SIPL1 gene amplification is able to differentiate BC from benign breast tissues (Fig 1B).

### Upregulation of SIPL1 mRNA in breast cancer

**Fig 2:**
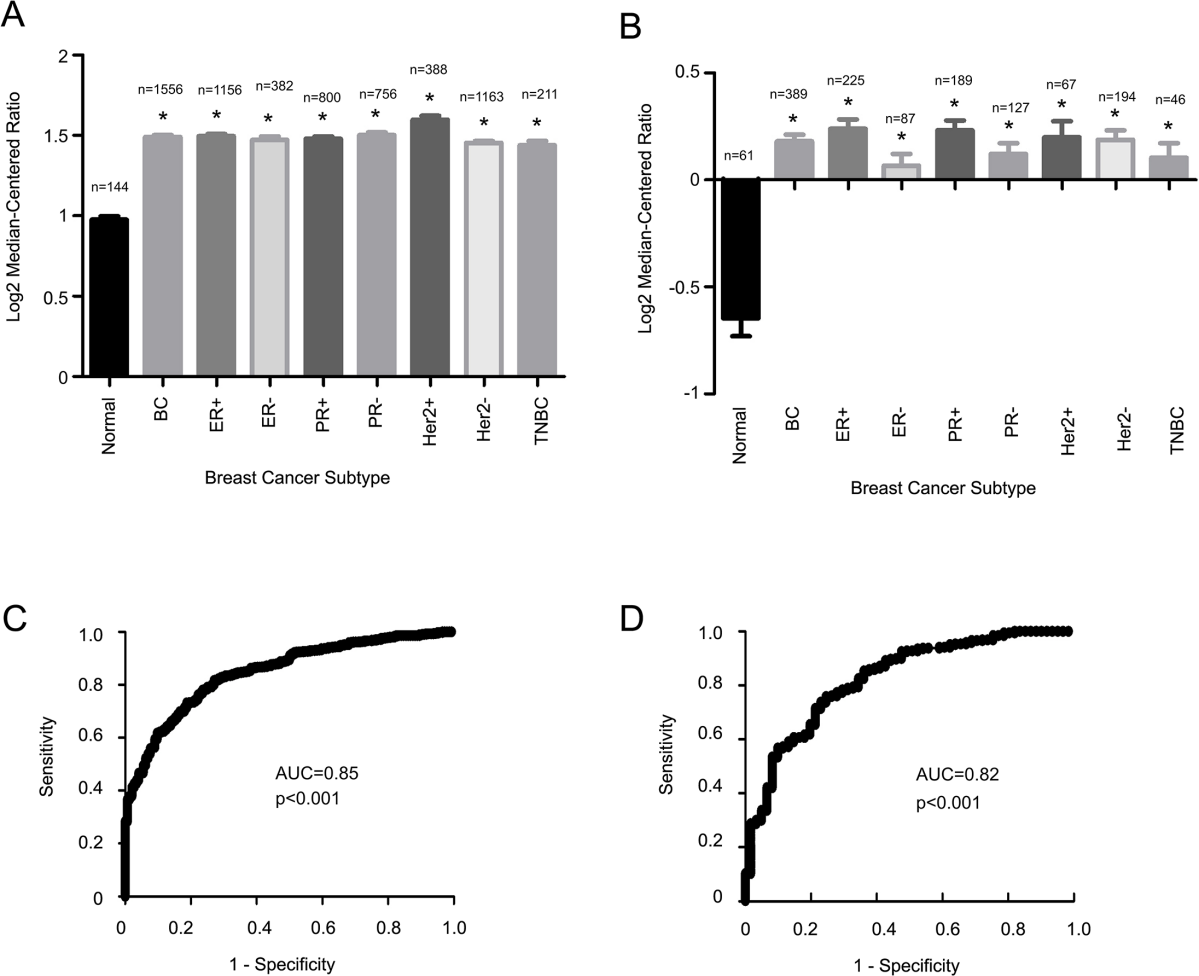
Increases in SIPL1 mRNA in breast cancer. Data from the Curtis (**A**) and TCGA datasets (**B**) were extracted from Oncomine and analyzed with respect to SIPL1 mRNA expression in cancer vs. normal tissues. Statistical Analysis was conducted using an unpaired, two-tailed, welch-corrected t-test. Asterisks indicate p<0.0001 in comparison the normal breast tissues. (C), (D) A receiver-operating characteristic (ROC) curve of normal versus primary breast cancer was calculated based the data extracted from the Curtis dataset (C) and the TCGA dataset (D).

The observed amplification of the *SIPL1* gene indicates that SIPL1 expression may be increased in breast cancer. To examine this scenario, we analyzed SIPL1 mRNA in 7 datasets available from Oncomine, in which both carcinoma and normal tissues are available (S1 Table). Increases in SIPL1 mRNA were observed in all breast cancer subtypes (ER+, PR+, HER2+, and triple negative BCs) in the two large datasets (Fig 2A and 2B). The elevation of SIPL1 mRNA levels in both datasets differentiated BC from normal breast tissues based on their respective ROC analysis (Fig 2C and 2D). Additionally, this upregulation was also largely detected in other smaller datasets (S1 Fig). Collectively, the above observations reveal an upregulation of SIPL1 mRNA in breast cancer.

### Elevation of SIPL1 expression correlates with breast cancer progression

Cancer progression is commonly measured by staging (Tumour stage I, II, III, IV) and grading (Grade 1, 2, 3) [36]. The above observations of increased *SIPL1* gene copy and upregulation of SIPL1 mRNA suggest that these alterations may associate with breast cancer progression. To determine this possibility, we analyzed the variations of the *SIPL1* gene copy number in three Oncomine datasets (Table 1). *SIPL1* gene copy number (GCN) increases associated with grading in the Curtis dataset but not in the Nikolsky dataset (Table 1). As the size of patient population in the Curtis dataset was more than 13 fold larger than it in the Nikolsky dataset, we preferred a positive correlation between an increase in *SIPL1* GCN and advancing BC grade. Although increases in *SIPL1* GCN were not significantly associated with BC staging in the Curtis dataset, a significant association could be established in the TCGA dataset (Table 1). A likely cause for this discrepancy is attributable to the limited number of stage III (n = 69) and stage IV (n = 9) tumors among the 1556 total cases in the Curtis dataset. Despite the TCGA dataset containing fewer than half of total BC cases in the Curtis dataset (Table 1), it had 138 stage III and 14 stage IV cases among its population of 639 tumours (Table 1). To attempt to compensate for the low number of advanced cases in the Curtis dataset, we performed a statistical analysis on stage I+II vs. stage III+IV cancers; this resulted in a decrease of the *p*-value from 0.437 (Table 1) to 0.1827. The same analysis also decreased the *p*-value from 0.016 (Table 1) to 0.0039 for the TCGA dataset. This analysis thus supports the likelihood that the Curtis dataset does not have a sufficient number of higher stage tumors to determine an association between increased *SIPL1* GCN and advancing BC stage. Collectively, the available evidence as a whole supports a correlation between *SIPL1* gene amplification and breast cancer progression.

**Table 1:** SIPL1 DNA copy number variation (CNV) in ductal breast carcinoma.

			Stage					Grade				
Dataset	BC^a^	Cases	I	II	III	IV	*p* ^c^	1	2	3	*p*	Ref.
Curtis 2	Ductal	1556	0.144 ^b^	0.159	0.196	0.214	0.4370	0.075	0.11	0.18	***<0*.*0001***	[58]
Nikolsky	Ductal	115						0.228	-0.029	0.057	0.2181	[66]
TCGA 2	Ductal	639	0.296	0.316	0.439	0.39	***0*.*016***					

^a^ Invasive ductal carcinoma

^b^ The Log2 copy number units reported within Oncomine, a Log2 copy number unit of 0 is converted to a gene copy number value of 2 according to the formula 2 X 2^(Log2 Copy Number Units)^

^c^ Statistical analysis was conducted using a One-Way ANOVA.

In accordance with this possibility, the examination of SIPL1 mRNA levels in 16 datasets of Oncomine containing 3127 cases (Table 2) indicates that upregulation of SIPL1 mRNA significantly associates with breast cancer staging and grading based on the data presented in the Curtis study, which is the largest dataset (Table 2). While a reduction of SIPL1 mRNA in stage IV breast cancers was observed in the largest dataset (Curtis), only 8 cases of stage IV tumors were included versus the large number of tumors of other stages (n = 257 for stage I, n = 446 for stage II, and n = 69 for stage III tumors) and called for precautious in interpretation of this decrease. Nonetheless, the results generally support a positive association between SIPL1 mRNA levels and breast cancer progression. This association is also consistent with the examination of the relationship between *SIPL1* gene copy number and breast cancer progression, in which amplification of the *SIPL1* gene associates with breast cancer grading within this same study (Table 1). Similar observations were also obtained using the TCGA datasets, as SIPL1 mRNA levels correlate with breast cancer staging (Table 2) as do the gains of the *SIPL1* gene (Table 1). However, this correlation was not observed in most of smaller studies (Table 2). Taken together, available data reveals a linkage between SIPL1 expression and breast cancer progression.

**Table 2:** SIPL1 mRNA Expression in Ductal Breast Carcinoma.

			Stage					Grade				
Dataset	BC^a^	Cases	I	II	III	IV	*p* ^c^	1	2	3	*p*	Ref.
Bittner	Ductal	161	0.918^b^	1.424	1.417	1.591	***0*.*0357***	0.729	1.304	1.445	***0*.*0016***	
Bonnefoi	Ductal	112						2.805	2.48	2.49	0.506	[67]
Curtis	Ductal	1556	1.396	1.493	1.528	1.396	***0*.*0198***	1.385	1.454	1.52	***0*.*0018***	[58]
Desmedt	Ductal	158						0.325	0.65	0.707	0.1221	[68]
Esserman	Ductal	93						-0.036	-0.33	-0.092	0.1261	[69]
Lu	Ductal	95						0.117	0.559	0.354	0.4078	[70]
Ma 3	Ductal	47						0.273	0.68	0.993	0.109	[71]
Pollack 2	Ductal	33						0.577	1.149	0.947	0.3438	[31]
Radvanyi	Ductal	30						1.594	2.084	1.795	0.5484	[72]
Sorlie	Ductal	65						0.349	0.954	0.99	0.1819	[24]
Sorlie 2	Ductal	90						-0.3	0.059	0.135	0.3348	[25]
Sotiriou 2	Ductal	97						0.37	0.316	0.291	0.5685	[73]
Tabchy	Ductal	163						0.738	0.714	0.668	0.6383	[74]
TCGA	Ductal	389	-0.043	0.171	0.276	0.151	0.0578					
Zhao	Ductal	38	*** ***	*** ***	*** ***	*** ***		-0.052	0.148	0.741	***0*.*0293***	[75]

^a^ Invasive ductal carcinoma

^b^ The Log2 Median-Centered Ratio reported within Oncomine

^c^ Statistical analysis was conducted using a One-Way ANOVA.

### SIPL1 expression predicts reduction in the survival of patients with ER+ or PR+ breast cancers

To consolidate the association of SIPL1 upregulation (gene copy number increases and mRNA elevation) with BC progression, we have analyzed the relationship between BC patients’ survival with either *SIPL1* gene copy number or SIPL1 mRNA levels using the Curtis dataset which contains 816 surviving patients with breast cancer and 429 patients who died from the disease (Table 3). In comparison to the survivors, those who died of breast cancer displayed a significantly higher gain of the *SILP1* gene and increase in the *SIPL1* mRNA (Table 3). Further analysis revealed that both increases in *SIPL1* GCN and mRNA level predicted worsening survival for patients with PR+ or ER+ but not those with HER2+ or triple negative BCs (Table 3).

**Table 3:** Differential Expression of SIPL1 correlates with survival in Ductal Breast Carcinoma within the Curtis dataset.

			SIPL1 mRNA level		SIPL1 gene copy number	
	Survivor	Deceased	Fold	*p*	Fold	*p*
**Overall**	816	429	1.088	***<0*.*0001***	1.255	***0*.*0162***
**ER+**	622	280	1.094	***<0*.*0001***	1.305	***0*.*0209***
**ER-**	183	147	1.082	***0*.*0116***	1.149	0.4066
**PR+**	449	180	1.108	***<0*.*0001***	1.343	0.0512
**PR-**	367	249	1.067	***0*.*0086***	1.139	0.2793
**Her2+**	184	134	1.045	0.1858	1.000	0.9976
**Her2-**	629	293	1.092	***<0*.*0001***	1.327	***0*.*0172***
**TNBC**	101	81	1.068	0.11054	1.070	0.7267

**Fig 3:**
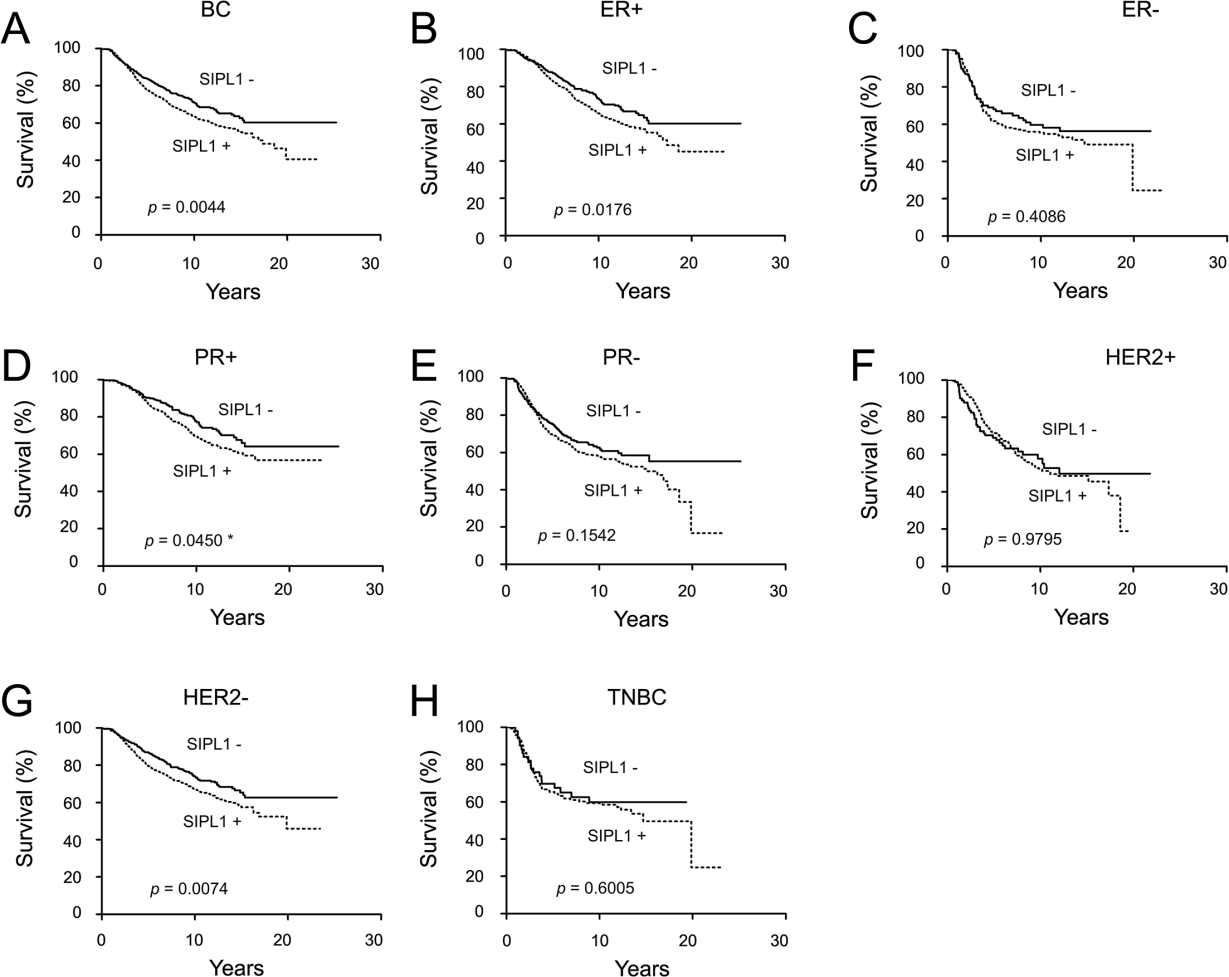
Increases in *SIPL1* gene copy number correlate with decreased survival for patients with breast cancer. Data was extracted from the Curtis dataset within Oncomine and analyzed with respect to gene copy number variation. Specifically, *SIPL1* copy number above 2 was labelled as SIPL1+ and those below 2 were indicated as SIPL1-. Kaplan–Meier analysis of survival for a subset of patients with *SIPL1* amplified breast cancer vs. those with breast cancer without *SIPL1* amplification (**A,** n = 1118 for *SIPL1*+ BCs, n = 127 for *SIPL1*- BCs), ER+ (**B,** n = 810 for *SIPL1*+ BCs, n = 92 for *SIPL1*- BCs), ER- (**C,** n = 295 for *SIPL1*+ BCs, n = 35 for *SIPL1*- BCs), PR+ (**D,** n = 565 for *SIPL1*+ BCs, n = 64 for *SIPL1*- BCs), PR- (**E,** n = 553 for *SIPL1*+ BCs, n = 63 for *SIPL1*- BCs), HER+ (**F,** n = 291 for *SIPL1*+ BCs, n = 27 for *SIPL1*- BCs), HER- (**G,** n = 822 for *SIPL1*+ BCs, n = 100 for *SIPL1*- BCs), and TNBC (**F,** n = 163 for *SIPL1*+ BCs, n = 21 for *SIPL1*- BCs). Only patients with follow up survival data were included. Any patients whose death was not related to the disease or with a non-specified cause were excluded. Statistical Analysis was conducted using a Log-Rank test. A *p*-value of <0.05 was considered statistically significant.

**Fig 4:**
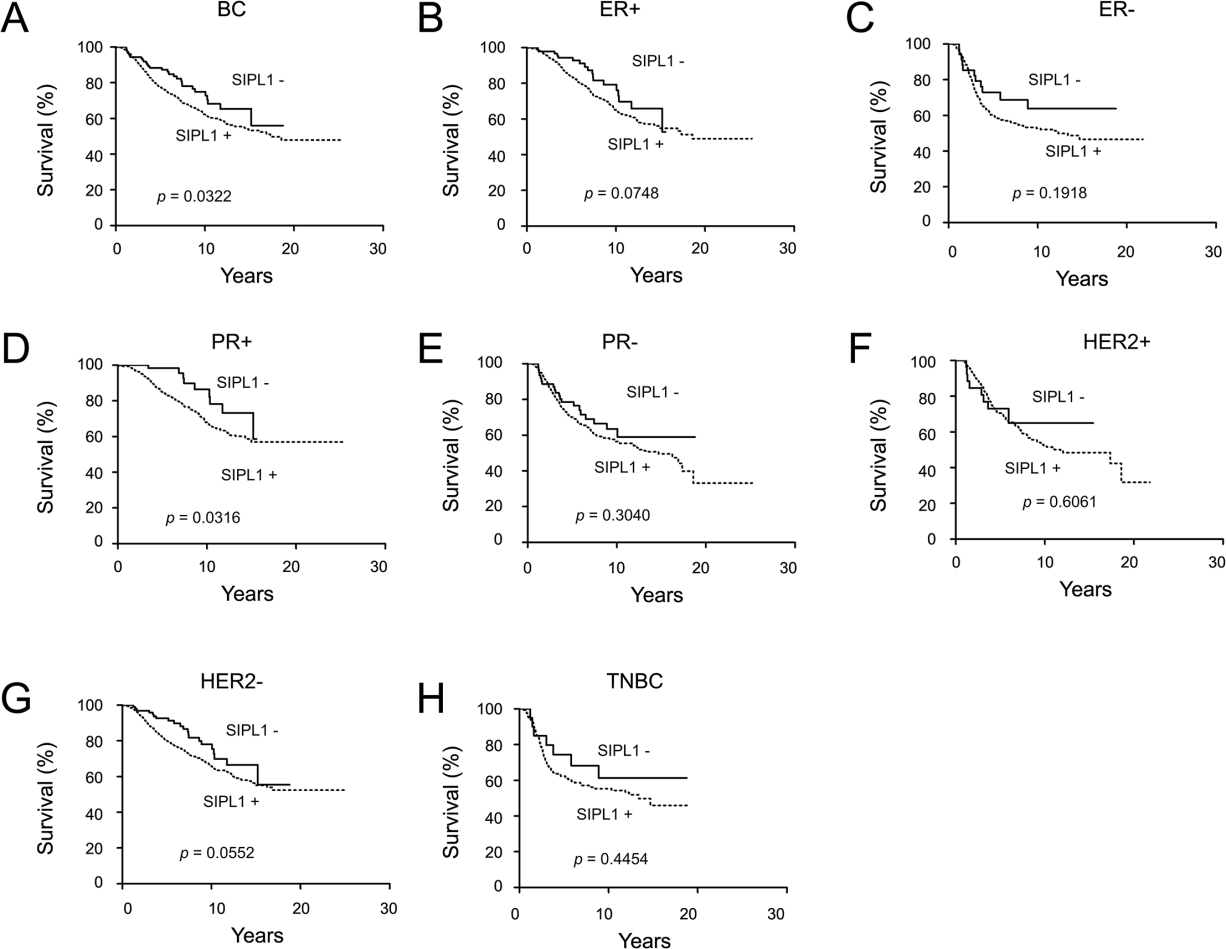
Increases in SIPL1 mRNA correlate with decreased survival for patients with breast cancer. Data was extracted from the Curtis dataset within Oncomine and analyzed with respect to gene copy number variation. Specifically, SIPL1 mRNA in normal breast tissues was averaged, which was used to determine if the cancer samples were with SIPL1 (positive) or without (negative) mRNA upregulation. Kaplan–Meier analysis of survival for a subset of patients with SIPL1+ amplified breast cancer vs those with SIPL1- breast cancer (**A,** n = 1039 for *SIPL1*+ BCs, n = 547 for *SIPL1*- BCs), ER+ (**B,** n = 751 for *SIPL1*+ BCs, n = 423 for *SIPL1*- BCs), ER- (**C,** n = 269 for *SIPL1*+ BCs, n = 111 for *SIPL1*- BCs), PR+ (**D,** n = 510 for *SIPL1*+ BCs, n = 306 for *SIPL1*- BCs), PR- (**E,** n = 529 for *SIPL1*+ BCs, n = 241 for *SIPL1*- BCs), HER+ (**F,** n = 258 for *SIPL1*+ BCs, n = 96 for *SIPL1*- BCs), HER- (**G,** n = 776 for *SIPL1*+ BCs, n = 451 for *SIPL1*- BCs), and TNBC (**F,** n = 168 for *SIPL1*+ BCs, n = 52 for *SIPL1*- BCs). Only patients with follow up survival data were included. Any patients whose death was not related to the disease or with a non-specified cause were excluded. Statistical Analysis was conducted using a Log-Rank test. A *p*-value of <0.05 was considered statistically significant.

This conclusion was further supported by our analysis of the time-to-decease endpoints (Kaplan-Meier analysis). Amplification of the *SIPL1* gene associates with decreased patient survival (Fig 3A), and this association is attributable to ER+ or PR+ breast cancers but not to those of HER2+ and triple negative (Fig 3B–3H). These results are in line with the linkage of SIPL1 mRNA levels with the decreased survival of breast cancer patients (Fig 4A) and in the patients with PR+ breast cancer (Fig 4D). The difference in survival of ER+ BC patients with elevated SIPL1 mRNA versus those without SIPL1 elevation approached statistical significance, but did not reach the 95% significance level (Fig 4B). This trend together with the observed association of *SIPL1* copy number increase with reducing survival of patients with ER+ BC (Fig 3B) support the notion that elevation of SIPL1 expression compromised the survival of patients with PR+ and ER+ breast cancer. Taken together, the above analyses demonstrate an inverse correlation of gain of the *SIPL1* gene or high levels of SIPL1 mRNA levels with decreasing survival in patients with PR+ or ER+ breast cancer.

### SIPL1 protein expression associates with ER+ and PR+ breast cancer

**Fig 5:**
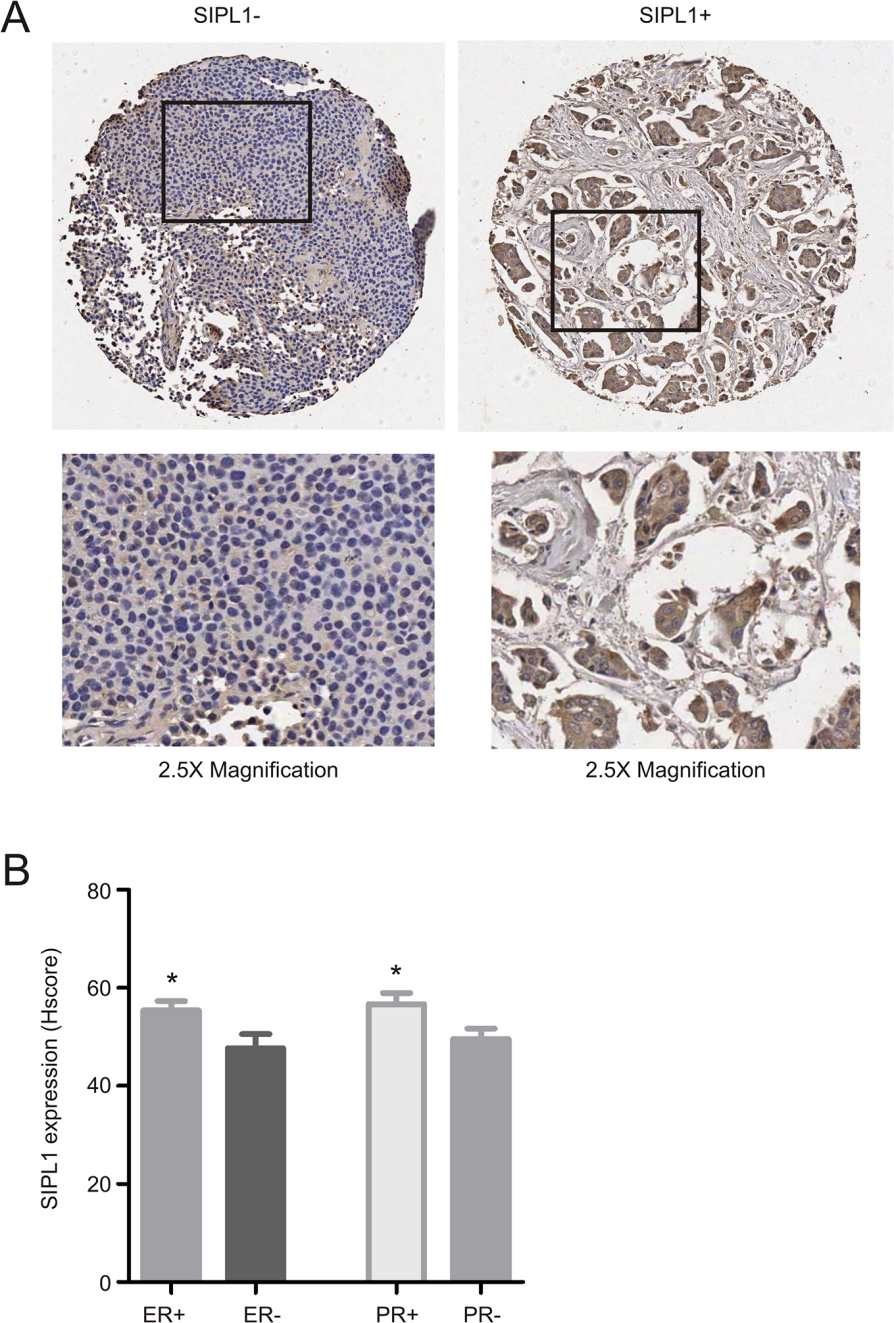
SIPL1 protein expression is associated with ER+ and PR+ tumours. (**A**) A TMA was examined for SIPL1 protein expression using IHC. Typical images of SIPL1+ and SIPL1- tumors are shown. The marked regions were enlarged 2.5 fold and placed underneath of the individual images. (**B**) SIPL1 staining was quantified (see Materials and Methods for details); means ± SEM (standard error mean) are graphed. * *p*<0.05 (unpaired, two-tailed, welch-corrected t-test).

To consolidate the above analyses using the Oncomine datasets, we examined SIPL1 protein expression by immunohistochemistry (IHC) using a tissue microarray containing 206 cases of primary breast cancers (S2 Table). IHC staining clearly detected the SIPL1 protein in some primary breast carcinomas (Fig 5). Quantification of SIPL1 staining by HScore detected higher levels of SIPL1 in ER+ and PR+ tumors versus those of ER- and PR- (Fig 5B). To examine whether SIPL1 expression correlates with ER+ or PR+ breast cancer, we divided the cancers into a group of strong SIPL1 expression (SIPL1+) and a group of weak SIPL1 expression (SIPL1-) based on an HScore of 40 (see Materials and Methods for justification). Fisher’s exact test revealed a correlation of SIPL1+ with ER+ and PR+ status (Table 4). These observations together with the association of increased *SIPL1* GCN and upregulation of the SIPL1 mRNA with reduction in the survival of patients with ER+ and PR+ breast cancer in the Oncomine datasets demonstrate a positive association between SIPL1 expression and ER+ and PR+ cancers. While SIPL1+ does not correlate with tumor size, tumor scores, node status, and metastasis, SIPL1+ correlates with tumor stage (Table 4), an observation that is consistent with the association of *SIPL1* gene copy number with BC stage detected in our analysis of the TCGA dataset (Table 1).

**Table 4:** The correlation of SIPL1 expression with the clinical-pathological parameters provided in the TMA.

	SIPL1 Expression		
	-	+	*p*-value
**Total (n = 206)**	33	173	
**Tumour Size**			
<20 mm	19	51	
>20 mm; <50 mm	28	86	Pearson r: 0.1632
>50 mm	8	14	0.7931 (Pearson’s Correlation)
**Tumour Stage**			
I	26	65	
II	21	68	
III	4	6	Pearson r: 0.9741
IV	4	12	***0*.*0259*** (Pearson’s Correlation)
**Node Status**			
N0	19	53	
N1+	28	71	0.8629 (Fisher’s exact test)
**Metastasis**			
M0	37	102	
M1	18	49	1.0000 (Fisher’s exact test)
**Tumour Score**			
1	6	26	
2	20	71	Pearson r: 0.1073
3	28	49	0.8637 (Pearson’s Correlation)
**Age**			
<50	15	39	
>50	40	112	0.8589 (Fisher’s exact test)
**ER**			
(+)	29	112	
(-)	26	38	***0*.*0037*** (Fisher’s exact test)
**PR**			
(+)	18	76	
(-)	37	74	***0*.*0268*** (Fisher’s exact test)
**AKT Activation**			
(-)	12	18	
(+)	42	133	0.0753 (Fisher’s exact test)

**Fig 6:**
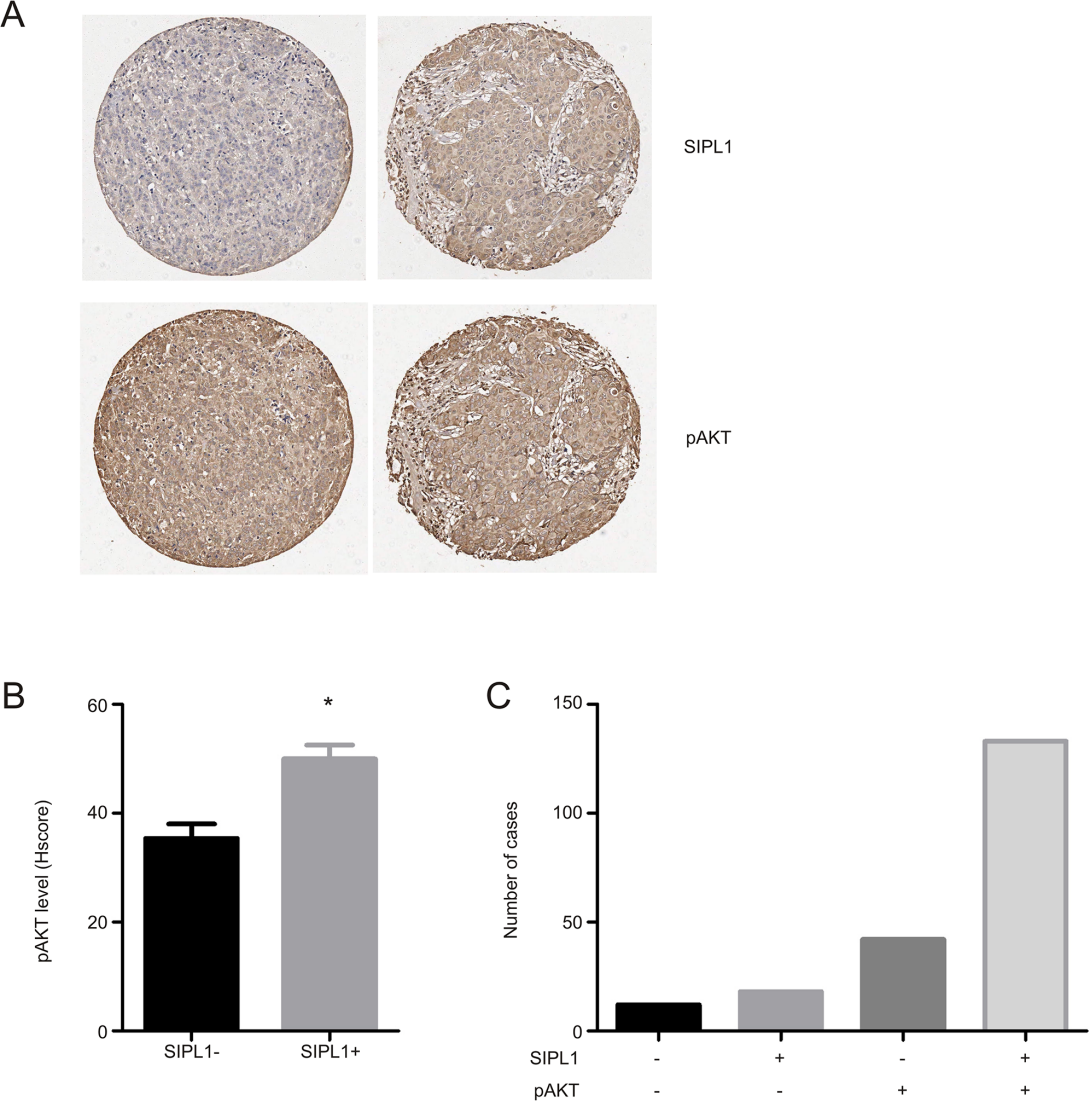
SIPL1 protein expression correlates with increased AKT activation in primary BC. (**A**) A TMA was examined for SIPL1 protein and Phospho-AKT Ser473 (pAKT). Typical images of tumors with pAKT in the presence of SIPL1+ and SIPL1- are shown. (**B**) AKT activation (pAKT) in SIPL1+ (n = 151) and SIPL1- (n = 54) was quantified; means ± SEM (standard error mean) are graphed. * *p*<0.05 (unpaired, two-tailed, welch-corrected t-test).

Supporting SIPL1's role in facilitating AKT activation in cervical cancer [20], we observed the co-existence of the SIPL1 protein with AKT activation in breast cancer (Fig 6A), although AKT activation was also detected in SIPL1- BC (Fig 6A). Quantification analysis revealed higher levels of AKT activation in SIPL1-positive breast cancer in comparison to those which were SIPL1-negative (Fig 6B). To examine a correlation of AKT activation with SIPL1 expression, in addition to the separation of BC into SIPL1+ and SIPL1- groups, BC were also catalogued into those of AKT+ or AKT- (see Materials and Methods for defining the threshold level). The co-existence of SIPL1+ and AKT+ (with AKT activation) in breast cancer could be evidently demonstrated (Fig 6C). Fisher’s exact test revealed a trend of correlation between SIPL1+ and AKT+ (Table 4).

### Progesterone negatively regulate SIPL1 expression

**Fig 7:**
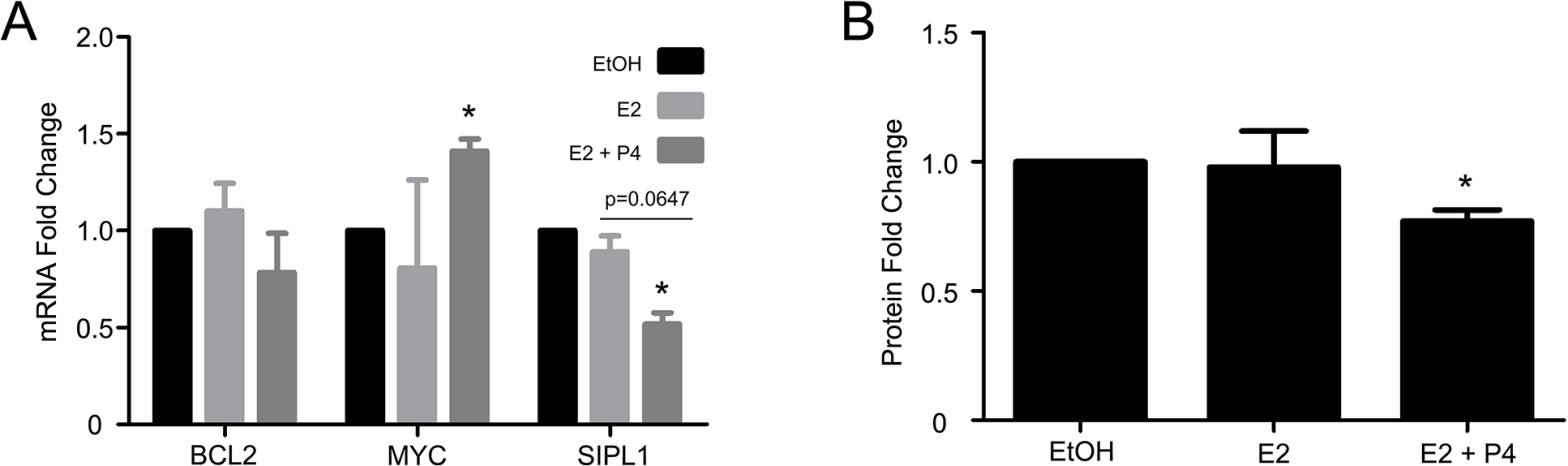
Progesterone reduces SIPL1 expression in MCF7 cells. (**A**) MCF7 cells cultured in estrogen and progesterone free conditions for 72 hours, treated with 10 nM E2 or a combination of 10 nM E2 and 10 nM P4 for 24 hours, and examined for changes in SIPL1 gene expression. The *BCL2* and *MYC* are E2 and P4 responsive respectively, and were used as positive controls for the treatments. * p<0.05 in comparison to the ethanol (Etoh) control (2-tailed student t-test). (**B**) Likewise, changes in SIPL1 protein expression were examined upon treatment with E2 or a combination of E2 and P4 (inset) and quantified using ImageJ. * p<0.05 in comparison to the ethanol (Etoh) control (2-tailed student t-test).

In view of the correlation of SIPL1 with ER and PR status observed above, we have analyzed the effects of estrogen and progesterone on SIPL1 expression. MCF7 cells are both ER and PR positive [37]. These cells were cultured in hormone free conditions for 72 hours, followed by stimulated with estrogen or estrogen plus progesterone. Because of estrogen-dependent PR expression, examination of PR-regulated genes was performed in the presence of estrogen. In comparison with estrogen alone, progesterone plus estrogen would identify genes whose expression is regulated by progesterone [38–41]. Both BCL2 and MYC genes are regulated by estrogen and progesterone, respectively [39,42–44]. As expected, estrogen showed a trend of BCL2 induction and progesterone significantly induced MYC expression (Fig 7A). Interestingly, addition of both estrogen and progesterone significantly downregulated SIPL1 mRNA, while estrogen alone had no effect (Fig 7A); this reduction was also confirmed at the protein level (Fig 7B). Collectively, these results are in line with the theme that SIPL1 plays a role in the tumorigenesis of PR+ breast cancer (see Discussion for details).

**Fig 8:**
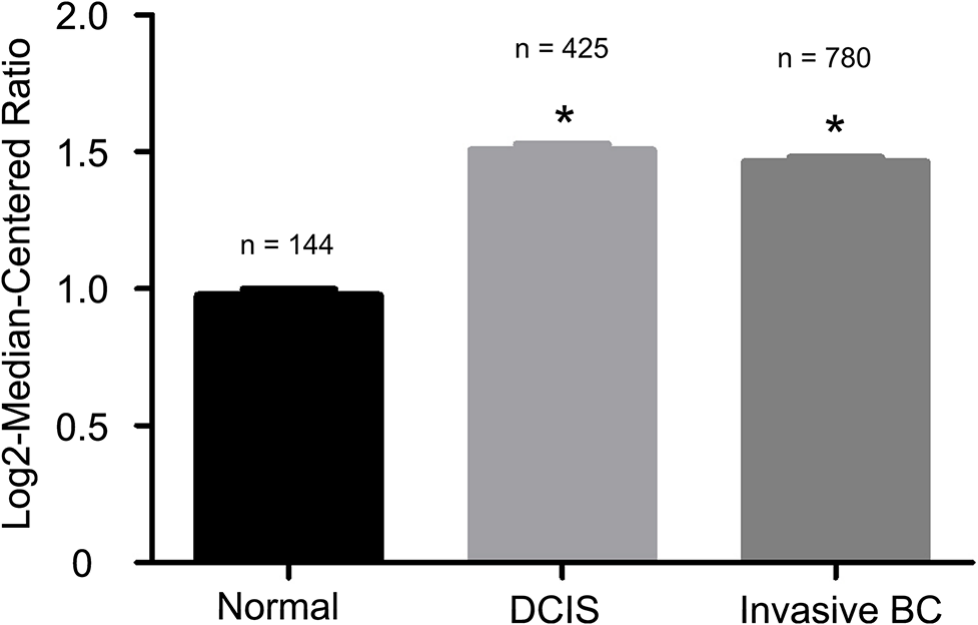
Comparison of SIPL1 mRNA expression in normal, DCIS and Invasive cancers. Utilizing the samples present in the Curtis dataset (Oncomine), the *SIPL1* gene expression level was compared between normal, DCIS (Stage 0) and Invasive tumors. Means ± SEM (standard error mean) are graphed. * p<0.05 (unpaired, two-tailed, welch-corrected t-test) compared to normal tissues.

We subsequently examined SIPL1 upregulation in the course of breast cancer tumorigenesis. Ductal carcinoma in situ (DCIS) is widely regarded as the precancerous lesion [45]. By taking advantage of the presence of 425 DCIS cases in the Curtis dataset (Oncomine), our analysis revealed a significant increase of SIPL1 mRNA in DCIS in comparison to normal breast tissue and that the SIPL1 mRNA levels remained high in invasive carcinoma (Fig 8), suggesting a critical role of SIPL1 in early stages of cancer formation.

## Discussion

Breast cancer is a heterogeneous disease, consisting of tumors expressing either ER or PR, which are the majority of breast cancers, and carcinomas classified as HER2+ or triple negative. It is well documented that the ER signalling plays an important role in the tumorigenesis of ER+ breast cancer, which is the scientific basis of the anti-estrogen therapy (tamoxifen and aromatase inhibitors). Recent evidence has also revealed that progesterone signalling plays an important role in promoting breast cancer tumorigenesis under specific conditions [46]. During the menstrual cycle, breast epithelial cells proliferate at the luteal phase, in which progesterone is at high levels [46,47]. In postmenopausal women undergoing hormone replacement therapy (HRT), the combination of estrogen with medroxyprogesterone acetate (MPA, a synthetic progestin) resulted in elevation of breast epithelial cell proliferation and breast density compared to those receiving estrogen alone [48]. High breast density, as detected by mammography, strongly associates with breast cancer risk [49–51]. In line with these discussions, the combination of estrogen and synthetic progestins increases breast cancer risk in postmenopausal women receiving HRT [52–54]. However, high levels of serum progesterone does not increase breast cancer risk in premenopausal women [55–57]. Collectively, evidence indicates that progesterone-associated risk of breast cancer depends on a woman’s age among other factors [46]. Nevertheless, the underlying mechanisms contributing to PR-facilitated breast cancer tumorigenesis remain essentially unclear.

Our analysis of the publicly available microarray datasets in Oncomine collectively demonstrates a common gain of the *SIPL1* gene and associated increases in SIPL1 mRNA expression in BC patients irrespective of receptor expression (Fig 1 and Fig 2). Comprehensive analysis of the largest dataset (1556 cases) of patients [58] revealed a reverse association between elevated SIPL1 expression with reduced patient survival in PR+ BC (Fig 3 and Fig 4). This possibility is supported by the linkage of the SIPL1 protein expression with PR+ BC observed in our own analysis of a BC TMA (Fig 5).

SIPL1 expression is also correlated with ER status. Gain of the *SIPL1* gene is associated with reduced survival for patients with ER+ breast cancer in our Oncomine analysis (Fig 3B). Likewise, a trend was observed in which high levels of SIPL1 mRNA was linked with poorer survival for ER+ BC patients (Fig 4B). Furthermore, our examination of SIPL1 protein expression demonstrated that SIPL1 associates with ER+ status (Fig 6B). Taken together, evidence supports a relationship between SIPL1 and ER+ BC.

The involvement of SIPL1 in the tumorigenesis of PR+ BCs was further supported by the progesterone-mediated downregulation of SIPL1 (Fig 7). These observations are intriguing considering the knowledge that PR signalling does not promote BC tumorigenesis in premenopausal women [55–57] and that PR functions differently in normal versus neoplastic tissues [59]. It is thus tempting to propose that suppression of SIPL1 may be a mechanism responsible for non-tumorigenic PR signalling and that SIPL1 upregulation may thus contribute to the removal of PR’s negative impact on BC tumorigenesis.

The correlation of SIPL1 expression with poor survival for patients with PR+ or ER+ breast cancer does not exclude the possible contributions of SIPL1 to the tumorigenesis of HER2+ and triple negative breast cancer, as gain of the *SIPL1* gene and increases in the SIPL1 mRNA were demonstrated in these BC types in comparison with normal breast tissues. Collectively, this investigation provides the first evidence of SIPL1 contributions to BC tumorigenesis.

While detailed mechanisms governing SIPL1-mediated BC tumorigenesis has yet to be elucidated, it is possible that multiple pathways may be involved. One of them is the induction of AKT activation (Fig 6), which is consistent with the reported role of SIPL1 in inhibiting PTEN activity [20]. Additionally, as a component of LUBAC, SIPL1/Sharpin activates NF-κB, which is known to promote tumorigenesis at multiple levels [60–62]. Despite this appealing mechanism, a PubMed search failed to uncover publications on the involvement of the LUBAC nor HOIL-1 or HOIP (two major components of the LUBAC) on breast cancer. Based on our analysis and work documented here, it will be intriguing to investigate whether LUBAC contributes to breast tumorigenesis. The recent characterization of LUBAC-mediated linear chain ubiquitination and the recently acquired knowledge of SIPL1/Sharpin structure will facilitate this investigation [63–65].

## Supporting Information

S1 Fig Alteration of SIPL1 mRNA in breast cancer. Data from the Perou (A), Randvanyi (**B**), Sorlie (**C**), Sorlie 2 (**D**) and Zhao (**E**) datasets were extracted from Oncomine and analyzed with respect to SIPL1 mRNA expression in cancer vs. normal tissues. Statistical Analysis was conducted using an unpaired, two-tailed, welch-corrected t-test. Asterisks indicate p<0.05 in comparison to normal breast tissues.
(TIF)

S1 Table Datasets used in the analysis of SIPL1 mRNA in breast cancers versus normal cases. (DOC)

S2 Table Clinical information for cases and their associated pAKT and SIPL1 Score. (DOC)
