# Supplementary figures and images for: Elevation of SIPL1 (SHARPIN) Increases Breast Cancer Risk
Source: PLoS One. 2015 May 19;10(5):e0127546. doi: 10.1371/journal.pone.0127546 (PMC4438068; doi:10.1371/journal.pone.0127546)

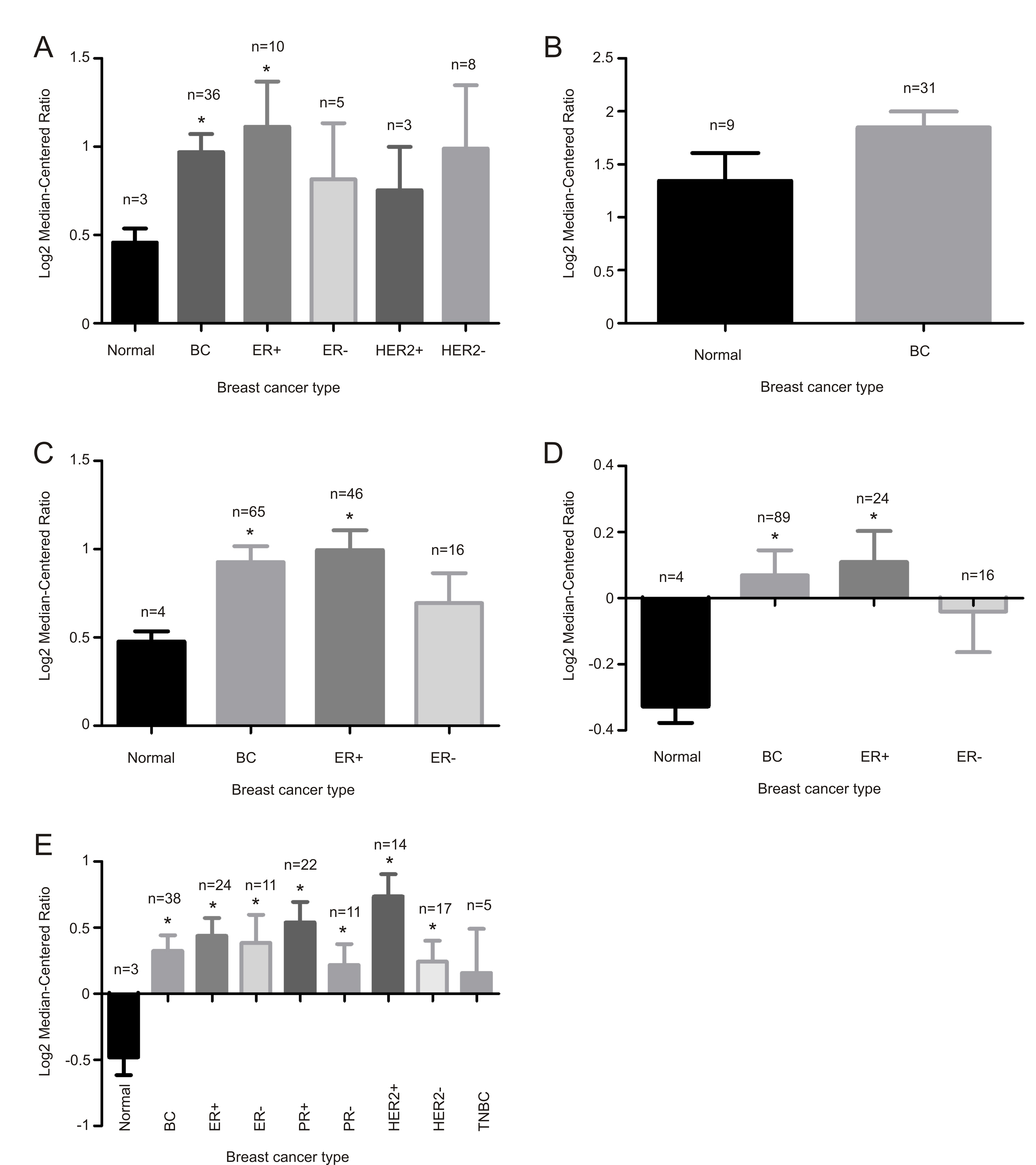

Supplement: S1 Fig — Data from the Perou (A), Randvanyi (B), Sorlie (C), Sorlie 2 (D) and Zhao (E) datasets were extracted from Oncomine and analyzed with respect to SIPL1 mRNA expression in cancer vs. normal tissues. Statistical Analysis was conducted using an unpaired, two-tailed, welch-corrected t-test. Asterisks indicate p<0.05 in comparison to normal breast tissues. (TIF) [file pone.0127546.s001.tif]
