# Supplementary material for: Elevation of SIPL1 (SHARPIN) Increases Breast Cancer Risk
Source: PLoS One. 2015 May 19;10(5):e0127546. doi: 10.1371/journal.pone.0127546 (PMC4438068; doi:10.1371/journal.pone.0127546)
Supplement: S1 Table — (DOC) [file pone.0127546.s002.doc]

**S1 Table^a^.**

Datasets used in the analysis of SIPL1 mRNA in breast cancers versus normal cases

| **Dataset** | **BC Type** | **Tumor Cases** | **Normal Cases** | **Ref.^b^** |
| --- | --- | --- | --- | --- |
| Curtis | Invasive Ductal BC | 1556 | 144 | [48] |
| Perou | Ductal BC | 36 | 3 | [65] |
| Radvanyi | Invasive Ductal BC | 30 | 9 | [61] |
| Sorlie | Ductal BC | 65 | 4 | [24] |
| Sorlie 2 | Ductal BC | 90 | 4 | [25] |
| TCGA | Invasive Ductal BC | 389 | 61 |  |
| Zhao | Invasive Ductal BC | 38 | 3 | [64] |
| **Total** |  | 2204 | 228 |  |

a: data from the indicted datasets were extracted from Oncomine (Compendia Bioscience,

Ann Arbor, MI)

b: references

BC: breast cancer
