# Supplementary material for: Elevation of SIPL1 (SHARPIN) Increases Breast Cancer Risk
Source: PLoS One. 2015 May 19;10(5):e0127546. doi: 10.1371/journal.pone.0127546 (PMC4438068; doi:10.1371/journal.pone.0127546)
Supplement: S2 Table — (DOC) [file pone.0127546.s003.doc]

**S2 Table.** Clinical information for cases and their associated pAKT and SIPL1 Score.

| **Case** | **Age** | **Stage** | **Node** | **Met** | **Tumour Size** | **Score** | **ER** | **PR** | **pAKT Lev** | **pAKT** | **SIPL1 Lev** | **SIPL1** |
| --- | --- | --- | --- | --- | --- | --- | --- | --- | --- | --- | --- | --- |
| 1 | 31 | T1C | N0 | M0 | 1.5 | 2 | 1 | 0 | 22.39 | 1 | 71.26 | 1 |
| 2 | 44 | T2 | N0 | M0 | 4.5 | 3 | 0 | 0 | 8.46 | 0 | 68.42 | 1 |
| 3 | 74 | T2 | N0 | M0 | 2.3 | 1 | 1 | 0 | 58.38 | 1 | 88.90 | 1 |
| 4 | 68 | T3 | NX | M1 | 6 | 3 | 0 | 0 | 24.06 | 1 | 65.20 | 1 |
| 5 | 53 | T3 | N1 | M1 | 5.3 | 3 | 1 | 1 | 32.49 | 1 | 68.82 | 1 |
| 6 | 53 | T2 | N1 | M1 | 2.8 | 2 | 1 | 1 | 58.04 | 1 | 99.59 | 1 |
| 7 | 67 | T1C | NX | M1 | 1.8 | 3 | 1 | 0 | 25.58 | 1 | 26.77 | 0 |
| 8 | 51 | T1C | N2 | M1 | 1.6 | 2 | 1 | 0 | 94.28 | 1 | 53.95 | 1 |
| 9 | 36 | T2 | N0 | M1 | 3 | 3 | 0 | 0 | 36.31 | 1 | 51.82 | 1 |
| 10 | 58 | T2 | N0 | M0 | 3.5 | 3 | 0 | 0 | 40.54 | 1 | 65.02 | 1 |
| 11 | 60 | T1 | N0 | M0 | 1.5 | 1 | 1 | 1 | 44.14 | 1 | 90.80 | 1 |
| 12 | 71 | T2 | N0 | M0 | 3.4 | 2 | 0 | 0 | 81.45 | 1 | 78.51 | 1 |
| 13 | 67 | T1C | N0 | M1 | 1.5 | 2 | 1 | 1 | 62.07 | 1 | 73.91 | 1 |
| 14 | 56 | T2 | N0 | M1 | 2.5 | 3 | 0 | 0 | 41.17 | 1 | 74.18 | 1 |
| 15 | 56 | T2 | N3 | M1 | 3.5 | 2 | 0 | 0 | 55.48 | 1 | 78.83 | 1 |
| 16 | 31 | T2 | N1 | M1 | 3 | 2 | 1 | 0 | 15.89 | 0 | 74.61 | 1 |
| 17 | 74 | T2 | N1 | M1 | 5 | 2 | 1 | 0 | 48.55 | 1 | 76.38 | 1 |
| 18 | 34 | T2 | N1 | M1 | 2.2 | 1 | 0 | 0 | 57.25 | 1 | 57.35 | 1 |
| 19 | 44 | T2 | NX | M1 | 3.5 | 1 | 1 | 1 | 85.33 | 1 | 82.90 | 1 |
| 20 | 75 | T1C | NX | M1 | 2 | 3 | 1 | 0 | 17.88 | 0 | 62.03 | 1 |
| 21 | 57 | T1 | N0 | M0 | 1 | 3 | 0 | 0 | 42.69 | 1 | 36.91 | 0 |
| 22 | 63 | T1C | N0 | M0 | 1.1 |  | 1 | 1 | 62.39 | 1 | 77.18 | 1 |
| 23 | 57 | T1C | N0 | M0 | 1.3 | 2 | 1 | 1 | 55.92 | 1 | 83.80 | 1 |
| 24 | 71 | T2 | N1 | M1 | 4 | 2 | 1 | 1 | 31.76 | 1 | 52.54 | 1 |
| 25 | 54 | T4B | N2 | M1 | 6 | 2 | 1 | 0 | 51.08 | 1 | 65.67 | 1 |
| 26 | 89 | T4B | NX | M1 | 9.5 | 2 | 1 | 1 | 66.17 | 1 | 104.49 | 1 |
| 27 | 84 | T3 | N1B | M1 | 5.5 | 3 | 1 | 0 | 60.68 | 1 | 61.21 | 1 |
| 28 | 64 | T2 | N1 | M1 | 3 | 3 | 1 | 0 | 13.79 | 0 | 27.72 | 0 |
| 29 | 70 | T2 | NX | M1 | 3.4 | 1 | 1 | 1 | 6.14 | 0 | 19.62 | 0 |
| 30 | 63 | T1C | NX | M1 | 2 | 2 | 1 | 1 | 84.89 | 1 | 81.37 | 1 |
| 31 | 71 | T2 | N0 | M0 | 2.5 | 1 | 1 | 0 | 51.33 | 1 | 81.81 | 1 |
| 32 | 52 | T4B | NX | M1 | 3.5 | 3 | 0 | 1 | 52.70 | 1 | 71.07 | 1 |
| 33 | 47 | T2 | N1 | M0 | 3.5 | 3 | 1 | 1 | 50.68 | 1 | 82.61 | 1 |
| 34 | 62 | T2 | N1 | M0 | 3.7 | 3 | 0 | 0 | 31.20 | 1 | 41.69 | 1 |
| 35 | 72 | T1B | N1 | M0 | 1 | 2 | 0 | 0 | 52.65 | 1 | 54.74 | 1 |
| 36 | 61 | T1 | N0 | M0 | 1.8 | 2 | 1 | 0 | 59.46 | 1 | 68.58 | 1 |
| 37 | 80 | T1C | N0 | M0 | 1.5 | 2 | 1 | 0 | 13.65 | 0 | 43.76 | 1 |
| 38 | 62 | T1C | N0 | M0 | 1.4 | 3 | 0 | 0 | 61.14 | 1 | 66.74 | 1 |
| 39 | 78 | T2 | N1 | M0 | 3.4 | 2 | 0 | 0 | 72.31 | 1 | 67.02 | 1 |
| 40 | 61 | T4B | N1 | M0 | 2 | 1 | 1 | 1 | 61.46 | 1 | 74.47 | 1 |
| 41 | 65 | T1C | N1 | M0 | 1.9 | 1 | 1 | 1 | 62.43 | 1 | 39.50 | 0 |
| 42 | 42 | T2 | N1B | M0 | 4 | 3 | 0 | 0 | 12.28 | 0 | 65.66 | 1 |
| 43 | 58 | T2 | N1B | M0 | 2.5 | 2 | 1 | 1 | 30.39 | 1 | 80.01 | 1 |
| 44 | 81 | T1C | N1 | M0 | 1.3 | 1 | 1 | NA | 63.53 | 1 | 123.05 | 1 |
| 45 | 44 | T1 | N1 | M0 | 1.5 | 2 | 0 | 0 | 58.95 | 1 | 65.23 | 1 |
| 46 | 70 | T2 | N1 | M0 | 3 | 1 | 1 | 0 | 98.97 | 1 | 70.18 | 1 |
| 47 | 50 | T1C | N0 | M0 | 1.2 | 2 | 1 | 0 | 19.42 | 0 | 37.68 | 0 |
| 48 | 55 | T1C | N0 | M0 | 2 | 1 | 1 | 1 | 77.43 | 1 | 88.02 | 1 |
| 49 | 80 | T1B | N0 | M0 | 1 | 2 | 1 | 1 | 77.82 | 1 | 119.23 | 1 |
| 50 | 32 | T1B | N1 | M0 | 1 | 3 | 0 | 0 | 50.70 | 1 | 44.36 | 1 |
| 51 | 44 | T2 | N1 | M0 | 2.3 | 2 | 0 | 0 | 59.60 | 1 | 108.67 | 1 |
| 52 | 53 | T1 | N1 | M0 | 1 | 1 | 0 | 0 | 58.61 | 1 | 62.67 | 1 |
| 53 | 56 | T1C | N1B | M0 | 1.7 | 3 | 1 | 0 | 44.44 | 1 | 90.33 | 1 |
| 54 | 66 | T1C | N0 | M0 | 1.5 | 2 | 1 | 1 | 53.15 | 1 | 70.97 | 1 |
| 55 | 68 | T1C | N0 | M0 | 1.8 | 1 | 1 | 1 | 71.62 | 1 | 68.63 | 1 |
| 56 | 83 | T2 | N0 | M0 | 2.2 | 1 | 1 | 0 | 65.88 | 1 | 72.93 | 1 |
| 57 | 69 | T1C | N0 | M0 | 1.8 | 2 | 1 | 1 | 67.36 | 1 | 54.09 | 1 |
| 58 | 68 | T2 | N1 | M0 | 5 | 1 | 0 | 1 | 27.21 | 1 | 22.62 | 0 |
| 59 | 65 | T2 | N1 | M0 | 4 | 3 | 0 | 0 | 30.80 | 1 | 82.36 | 1 |
| 60 | 63 | T2 | N1 | M0 | 3.5 | 3 | 0 | 0 | 52.24 | 1 | 73.05 | 1 |
| 61 | 74 | T1C | N1 | M0 | 1.2 | 2 | 1 | 1 | 35.73 | 1 | 51.91 | 1 |
| 62 | 72 | T2 | N1B2 | M0 | 3 | 2 | 1 | 1 | 75.65 | 1 | 78.52 | 1 |
| 63 | 60 | T1C | N0 | M0 | 1.9 | 1 | 1 | 1 | 37.93 | 1 | 67.64 | 1 |
| 64 | 51 | T1 | N0 | M0 | 1.5 | 2 | 1 | 1 | 62.39 | 1 | 101.40 | 1 |
| 65 | 49 | T2 | N1B | M0 | 2.5 | 2 | 0 | 0 | 68.82 | 1 | 98.42 | 1 |
| 66 | 40 | T3 | N1 | M1 | 8 | 3 | 0 | 0 | -33.35 | 0 | 28.37 | 0 |
| 67 | 53 | T2 | N1B | M1 | 4 | 2 | 1 | 0 | 59.31 | 1 | 40.18 | 1 |
| 68 | 72 | T2 | NX | M1 | 2.5 | 3 | 1 | 1 | 39.56 | 1 | 56.98 | 1 |
| 69 | 59 | T1 | N0 | M0 | 1.4 | 2 | 1 | 1 | 45.18 | 1 | 40.14 | 1 |
| 70 | 59 | T2 | N0 | M0 | 2.6 | 3 | 0 | 0 | 36.80 | 1 | 29.48 | 0 |
| 71 | 72 | T1 | N1 | M0 | 1.8 | 1 | 1 | 1 | 55.08 | 1 | 42.97 | 1 |
| 72 | 52 | T2 | N1B | M0 | 2.2 | 3 | 0 | 0 | 25.86 | 1 | 24.23 | 0 |
| 73 | 70 | T2 | N1 | M0 | 5 | 1 | 1 | 0 | 31.71 | 1 | 47.36 | 1 |
| 74 | 44 | T1C | N1 | M0 | 2 | 2 | 0 | 1 | 24.24 | 1 | 51.78 | 1 |
| 75 | 46 | T2 | N1A | M1 | 5 | 2 | 1 | 0 | 20.54 | 1 | 1.68 | 0 |
| 76 | 78 | T2 | N2 | M1 | 3.5 | 3 | 0 | 0 | 31.08 | 1 | 42.08 | 1 |
| 77 | 64 | T2 | N2 | M1 | 3.5 | 3 | 0 | 1 | 34.25 | 1 | 36.26 | 0 |
| 78 | 56 | T1B | NX | M1 | 1 | 2 | 1 | 0 | 40.47 | 1 | 53.65 | 1 |
| 79 | 35 | T1C | N0 | M0 | 2 | 3 | 0 | 0 | 36.11 | 1 | 9.99 | 0 |
| 80 | 49 | T2 | N0 | M0 | 3 | 2 | 0 | 1 | 45.50 | 1 | 41.27 | 1 |
| 81 | 53 | T1 | N0 | M0 | 1 | 1 | 1 | 1 | 39.71 | 1 | 38.99 | 0 |
| 82 | 49 | T1B | N0 | M0 | 1 | 1 | 1 | 1 | 36.47 | 1 | 44.76 | 1 |
| 83 | 36 | T2 | N1 | M0 | 4.8 | 3 | 0 | 0 | 27.82 | 1 | 27.28 | 0 |
| 84 | 38 | T1C | N1 | M0 | 1.7 | 2 | 0 | 0 | 80.83 | 1 | 56.86 | 1 |
| 85 | 64 | T1C | N1 | M0 | 1.5 | 3 | 0 | 0 | 42.95 | 1 | 41.15 | 1 |
| 86 | 83 | T1C | N1 | M0 | 1.2 | 2 | 0 | 0 | 54.10 | 1 | 28.00 | 0 |
| 87 | 43 | T3 | NX | M1 | 7 | 2 | 1 | 1 | 54.61 | 1 | 28.57 | 0 |
| 88 | 74 | T3 | N1 | M1 | 5.5 | 3 | 1 | 0 | 33.57 | 1 | 1.51 | 0 |
| 89 | 83 | T2 | NX | M1 | 3.5 | 2 | 1 | 1 | 13.92 | 0 | 11.37 | 0 |
| 90 | 63 | T2 | NX | M1 | 2.5 | 3 | 1 | 1 | 40.36 | 1 | 34.06 | 0 |
| 91 | 59 | T1C | N0 | M0 | 2 | 3 | 0 | 0 | 23.00 | 1 | 8.52 | 0 |
| 92 | 78 | T1C | N0 | M0 | 2 | 2 | 1 | 1 | 62.88 | 1 | 23.01 | 0 |
| 93 | 81 | T2 | N0 | M0 | 3 | 3 | 1 | 1 | 25.73 | 1 | 35.09 | 0 |
| 94 | 43 | T1C | N1 | M0 | 2 | 2 | 1 | 1 | 47.72 | 1 | 28.66 | 0 |
| 95 | 67 | T1C | N1 | M0 | 2 | 2 | 1 | 0 | 45.58 | 1 | 55.64 | 1 |
| 96 | 71 | T2 | N1 | M0 | 3 | 3 | 0 | 0 | 20.06 | 1 | 12.86 | 0 |
| 97 | 51 | T1 | N1 | M1 | 1.8 | 2 | 0 | 0 | 16.51 | 0 | 29.34 | 0 |
| 98 | 73 | T2 | N0 | M0 | 3 | 1 | 1 | 1 | 91.03 | 1 | 78.48 | 1 |
| 99 | 32 | T2 | N1 | M0 | 2.4 | 3 | 0 | 0 | 27.17 | 1 | 23.91 | 0 |
| 100 | 89 | T2 | N0 | M0 | 2.5 | 2 | 1 | 1 | 35.75 | 1 | 45.82 | 1 |
| 101 | 41 | T1C | N0 | M0 | 1.5 | 3 | 0 | 0 | 19.87 | 0 | 9.08 | 0 |
| 102 | 63 | T1C | N0 | M0 | 2 | 2 | 0 | 0 | 47.98 | 1 | 36.94 | 0 |
| 103 | 51 | T1 | N0 | M0 | 1.5 | 2 | 1 | 1 | 32.97 | 1 | 34.88 | 0 |
| 104 | 55 | T2 | N1 | M0 | 3.1 | 3 | 0 | 0 | 25.31 | 1 | 12.91 | 0 |
| 105 | 78 | T1C | N1 | M0 | 1.5 | 2 | 1 | 1 | 42.73 | 1 | 45.19 | 1 |
| 106 | 44 | T2 | N1B | M0 | 4 | 2 | 1 | 1 | 74.53 | 1 | 21.90 | 0 |
| 107 | 40 | T2 | N1 | M0 | 4.9 | 3 | 1 | 1 | 47.55 | 1 | 47.29 | 1 |
| 108 | 85 | T4B | N1 | M1 | 3 | 2 | 1 | 1 | 84.81 | 1 | 56.35 | 1 |
| 109 | 43 | T2 | N1 | M1 | 3.5 | 2 | 1 | 0 | 38.11 | 1 | 40.76 | 1 |
| 110 | 70 | T3 | N1 | M1 | 10 | 2 | 1 | 0 | 21.44 | 1 | 39.83 | 0 |
| 111 | 59 | T2 | N1 | M1 | 2.5 | 2 | 1 | 0 | 5.67 | 0 | 51.95 | 1 |
| 112 | 40 | T1C | N0 | M0 | 1.2 | 3 | 0 | 1 | 35.45 | 1 | 28.63 | 0 |
| 113 | 41 | T1C | N0 | M0 | 1.6 | 1 | 1 | 1 | 55.97 | 1 | 40.40 | 1 |
| 114 | 47 | T2 | N0 | M0 | 3 | 3 | 0 | 0 | 48.45 | 1 | 24.51 | 0 |
| 115 | 70 | T2 | N0 | M0 | 4.3 | 2 | 1 | 1 | 58.09 | 1 | 41.69 | 1 |
| 116 | 83 | T1C | N1 | M0 | 1.5 | 2 | 1 | 1 | 67.43 | 1 | 34.22 | 0 |
| 117 | 70 | T1C | N1 | M0 | 1.8 | 3 | 0 | 0 | 45.36 | 1 | 20.85 | 0 |
| 118 | 70 | T2 | N1 | M0 | 3 | 3 | 1 | 1 | 84.47 | 1 | 40.11 | 1 |
| 119 | 63 | T2 | N1 | M0 | 4.6 | 2 | 1 | 1 | 43.41 | 1 | 41.08 | 1 |
| 120 | 48 | T2 | NX | M1 | 2.7 | 3 | 1 | 1 | 43.46 | 1 | 55.12 | 1 |
| 121 | 82 | T1C | N0 | M0 | 1.8 | 1 | 1 | 1 | 2.80 | 0 | 38.37 | 0 |
| 122 | 46 | T1C | N0 | M0 | 1.5 |  | 0 | 0 | -74.84 | 0 | 27.36 | 0 |
| 123 | 67 | T2 | N0 | M0 | 3 | 3 | 1 | 0 | 12.67 | 0 | 40.27 | 1 |
| 124 | 66 | T1B | N0 | M0 | 1 | 3 | 1 | 1 | 62.91 | 1 | 17.01 | 0 |
| 125 | 61 | T2 | N1 | M0 | 2.5 | 2 | 1 | 1 | 66.77 | 1 | 54.44 | 1 |
| 126 | 41 | T1 | N1 | M0 | 1.5 | 2 | 0 | 0 | 56.04 | 1 | 47.44 | 1 |
| 127 | 41 | T4 | N2 | M0 | 4.5 | 3 | 0 | 0 | 57.43 | 1 | 23.88 | 0 |
| 128 | 87 | T2 | N1 | M0 | 4.5 | 3 | 0 | 0 | 69.18 | 1 | 25.39 | 0 |
| 129 | 77 | T1C | NX | M1 | 1.8 |  | 1 | 0 | 122.07 | 1 | 63.78 | 1 |
| 130 | 62 | T1C | NX | M1 | 1.5 | 2 | 1 | 0 |  |  | -57.45 | 0 |
| 131 | 35 | T2 | NX | M1 | 3 | 3 | 1 | 1 | 143.16 | 1 | 63.13 | 1 |
| 132 | 58 | T4 | N2 | M1 | 3.5 | 3 | 1 | 0 | 39.51 | 1 | 54.55 | 1 |
| 133 | 51 | T2 | N0 | M0 | 2.3 | 3 | 0 | 0 | -13.53 | 0 | 46.65 | 1 |
| 134 | 58 | T1C | N2 | M0 | 2 | 3 | 1 | 0 | 54.08 | 1 | 36.78 | 0 |
| 135 | 85 | T1C | NX | M1 | 1.1 | 2 | 1 | 1 | 70.79 | 1 | 52.05 | 1 |
| 136 | 68 | T1B | N1 | M0 | 1 | 2 | 1 | 1 | 57.05 | 1 | 58.69 | 1 |
| 137 | 45 | T1C | N1 | M0 | 1.5 | 2 | 1 | 1 | 26.66 | 1 | 66.37 | 1 |
| 138 | 56 | T2 | N1B | M0 | 3 |  | 1 | 1 | 10.95 | 0 | 45.61 | 1 |
| 139 | 41 | T2 | N1B | M0 | 4 | 3 | 1 | 1 | 8.41 | 0 | 45.16 | 1 |
| 140 | 37 | T4 | N2 | M1 | 6.5 | 3 | 0 | 0 | 13.58 | 0 | 42.62 | 1 |
| 141 | 58 | T1C | NX | M1 | 1.5 | 2 | 1 | 0 | 13.46 | 0 | 41.66 | 1 |
| 142 | 88 | T3 | NX | M1 | 6 | 3 | 1 | 0 | 18.89 | 0 | 47.52 | 1 |
| 143 | 77 | T1C | NX | M1 | 2 | 2 | 1 | 1 | 50.51 | 1 | 54.73 | 1 |
| 144 | 41 | T1C | N0 | M0 | 1.2 | 2 | 1 | 1 | 75.91 | 1 | 72.30 | 1 |
| 145 | 44 | T2 | N0 | M0 | 4.5 | 3 | 0 | 0 | 12.72 | 0 | 48.03 | 1 |
| 146 | 67 | T2 | N0 | M0 | 2.4 | 3 | 1 | 0 | 68.21 | 1 | 83.06 | 1 |
| 147 | 55 | T2 | N0 | M0 | 4 | 3 | 0 | 0 | 25.87 | 1 | 65.14 | 1 |
| 148 | 63 | T2 | N2 | M0 | 3.5 | 3 | 1 | 0 | 24.55 | 1 | 41.10 | 1 |
| 149 | 61 | T2 | N1 | M0 | 2.5 | 1 | 1 | 0 | 37.91 | 1 | 80.27 | 1 |
| 150 | 65 | T2 | N1 | M0 | 3.7 | 2 | NA | 1 | 58.83 | 1 | 90.85 | 1 |
| 151 | 79 | T2 | N1B | M0 | 3 | 3 | 1 | 1 | 23.28 | 1 | 55.02 | 1 |
| 152 | 71 | T2 | NX | M1 | 2.1 | 2 | 1 | 0 | 29.00 | 1 | 59.91 | 1 |
| 153 | 62 | T4C | NX | M1 | 9 | 2 | 1 | 1 | 32.56 | 1 | 48.86 | 1 |
| 154 | 59 | T1C | NX | M1 | 1.5 | 2 | 0 | 0 | 40.19 | 1 | 62.50 | 1 |
| 155 | 70 | T1C | N0 | M1 | 1.2 | 1 | 1 | 1 | 54.63 | 1 | 63.23 | 1 |
| 156 | 52 | T1C | N0 | M0 | 2 | 2 | 1 | 1 | 26.01 | 1 | 41.24 | 1 |
| 157 | 32 | T1C | N0 | M0 | 1.3 | 3 | 1 | 1 | 24.19 | 1 | 63.23 | 1 |
| 158 | 71 | T1 | N0 | M0 | 1.8 | 1 | 1 | 1 | 30.87 | 1 | 74.84 | 1 |
| 159 | 39 | T1C | N1 | M0 | 2 | 1 | 1 | 1 | 19.78 | 0 | 53.48 | 1 |
| 160 | 51 | T2 | N1 | M0 | 3 | 2 | 0 | 0 | 21.30 | 1 | 34.91 | 0 |
| 161 | 43 | T2 | N1 | M0 | 3.8 | 3 | 1 | 0 | 39.64 | 1 | 76.75 | 1 |
| 162 | 70 | T1C | NX | M1 | 2 | 3 | 1 | 1 | 87.94 | 1 | 66.06 | 1 |
| 163 | 66 | T4B | N1 | M1 | 3.7 | 3 | 1 | 0 | 22.72 | 1 | 36.40 | 0 |
| 164 | 75 | T2 | N1B1 | M1 | 5 | 2 | 1 | 1 | 21.38 | 1 | 28.85 | 0 |
| 165 | 60 | T1B | N0 | M0 | 1 | 2 | 1 | 0 | 8.93 | 0 | 28.31 | 0 |
| 166 | 75 | T2 | N0 | M0 | 2.5 |  | 1 | 1 | 46.76 | 1 | 52.50 | 1 |
| 167 | 58 | T1 | N0 | M0 | 1.5 | 2 | 1 | 1 | 35.98 | 1 | 50.20 | 1 |
| 168 | 56 | T1C | N1 | M0 | 1.8 | 2 | 1 | 0 | 20.92 | 1 | 68.92 | 1 |
| 169 | 67 | T1B | NX | M1 | 1 | 2 | 1 | 1 | 19.37 | 0 | 47.53 | 1 |
| 170 | 53 | T1 | N0 | M0 | 2 | 3 | 0 | 0 | 29.17 | 1 | 68.58 | 1 |
| 171 | 70 | T3 | NX | M1 | 6 | 1 | 1 | 1 | 44.82 | 1 | 75.08 | 1 |
| 172 | 84 | T2 | NX | M1 | 2.5 | 3 | 1 | 1 | 17.87 | 0 | 43.31 | 1 |
| 173 | 64 | T4 | N1 | M1 | 3.7 | 2 | 0 | 0 | 46.36 | 1 | 67.23 | 1 |
| 174 | 54 | T4D | NX | M1 | 3.5 | 2 | 1 | 0 | 45.38 | 1 | 87.57 | 1 |
| 175 | 36 | T2 | N0 | M0 | 3.5 | 3 | 1 | 0 | 32.91 | 1 | 49.15 | 1 |
| 176 | 75 | T1C | N0 | M0 | 1.2 | 2 | 1 | 1 | 48.24 | 1 | 41.71 | 1 |
| 177 | 62 | T1 | N0 | M0 | 2 | 3 | 0 | 0 | 21.12 | 1 | 35.61 | 0 |
| 178 | 69 | T1C | N0 | M0 | 1.5 | 2 | 1 | 0 | 22.83 | 1 | 43.02 | 1 |
| 179 | 52 | T2 | N1 | M0 | 2.7 | 3 | 1 | 1 | 24.39 | 1 | 41.34 | 1 |
| 180 | 26 | T2 | N1 | M0 | 3 | 3 | 0 | 1 | 36.70 | 1 | 54.90 | 1 |
| 181 | 68 | T2 | N2 | M0 | 3 | 2 | 1 | 1 | 54.65 | 1 | 69.39 | 1 |
| 182 | 66 | T2 | N1 | M0 | 2.5 | 1 | 1 | 1 | 81.59 | 1 | 79.95 | 1 |
| 183 | 81 | T1C | NX | M1 | 1.6 | 2 | 0 | 1 | 64.91 | 1 | 107.44 | 1 |
| 184 | 71 | T2 | NX | M1 | 2.5 | 1 | 1 | 0 | 74.62 | 1 | 79.70 | 1 |
| 185 | 44 | T1C | N0 | M0 | 1.5 | 2 | 1 | 1 | 68.13 | 1 | 89.44 | 1 |
| 186 | 70 | T1 | N0 | M0 | 2 | 3 | 1 | 0 | 46.25 | 1 | 58.81 | 1 |
| 187 | 53 | T2 | N0 | M0 | 4.5 | 2 | 1 | 0 | 26.47 | 1 | 33.95 | 0 |
| 188 | 57 | T1C | N1B | M0 | 1.2 | 2 | 1 | 0 | 36.97 | 1 | 55.10 | 1 |
| 189 | 69 | T1C | N1 | M0 | 1.5 | 1 | 1 | 0 | 35.77 | 1 | 34.99 | 0 |
| 190 | 64 | T1C | N1 | M0 | 2 | 3 | 0 | 0 | 44.14 | 1 | 43.94 | 1 |
| 191 | 59 | T2 | N1 | M0 | 5 |  | 0 | 0 | 79.10 | 1 | 50.54 | 1 |
| 192 | 47 | T1C | NX | M1 | 2 | 2 | 1 | 1 | 35.35 | 1 | 55.20 | 1 |
| 193 | 76 | T4 | NX | M1 | 18 | 2 | 1 | 1 | 29.85 | 1 | 58.85 | 1 |
| 194 | 33 | T1B | NX | M1 | 1 | 3 | 0 | 0 | 12.47 | 0 | 38.48 | 0 |
| 195 | 61 | T4 | NX | M1 | 6 | 2 | 1 | 0 | 18.61 | 0 | 34.51 | 0 |
| 196 | 41 | T2 | N0 | M0 | 4.1 | 3 | 1 | 0 | 51.68 | 1 | 56.01 | 1 |
| 197 | 37 | T2 | N0 | M0 | 2.8 | 3 | 1 | 0 | 38.90 | 1 | 47.78 | 1 |
| 198 | 87 | T1C | N0 | M0 | 1.3 | 1 | 1 | 1 | 44.61 | 1 | 53.63 | 1 |
| 199 | 39 | T1C | N0 | M0 | 2 | 2 | 1 | 1 | 53.04 | 1 | 71.73 | 1 |
| 200 | 63 | T4 | N1 | M0 | 4.5 | 3 | 1 | 1 | 43.30 | 1 | 63.95 | 1 |
| 201 | 73 | T2 | N1 | M0 | 3.7 | 3 | 0 | 0 | 34.01 | 1 | 37.57 | 0 |
| 202 | 34 | T1C | N1B | M0 | 1.5 | 2 | 1 | 1 | 23.27 | 1 | 49.62 | 1 |
| 203 | 31 | T3 | N1 | M0 | 6 | 2 | 1 | 0 | 37.03 | 1 | 48.98 | 1 |
| 204 | 58 | T4 | N1 | M1 | 4 | 2 | 1 | 0 | 51.53 | 1 | 39.97 | 0 |
| 205 | 53 | T2 | N0 | M0 | 2.3 | 3 | 1 | 1 | 21.43 | 1 | 40.56 | 1 |
| 206 | 60 | T2 | N1 | M0 | 2.8 | 2 | 1 | 0 | 14.77 | 0 | 40.77 | 1 |

Age: at diagnosis, pAKT Lev: pAKT HScores; SILP1 Lev: SIPL1 HScores; ER, PR: 1-positive, 0-negative; pAKT, SIPL1: 1-positive, 0-negative (see Materials and Methods for the conversion based on the respective HScores).
